# The *recombination activation gene 1 *(*Rag1*) is expressed in a subset of zebrafish olfactory neurons but is not essential for axon targeting or amino acid detection

**DOI:** 10.1186/1471-2202-6-46

**Published:** 2005-07-15

**Authors:** Bo Feng, Sarada Bulchand, Emre Yaksi, Rainer W Friedrich, Suresh Jesuthasan

**Affiliations:** 1 Developmental Neurobiology Group, Temasek LifeSciences Laboratory, 1 Research Link, The National University of Singapore, 117604, Singapore; 2 Max Planck Institute for Medical Research, Dept. of Biomedical Optics, Jahnstr. 29, D-69120 Heidelberg, Germany

## Abstract

**Background:**

*Rag1 *(Recombination activation gene-1) mediates genomic rearrangement and is essential for adaptive immunity in vertebrates. This gene is also expressed in the olfactory epithelium, but its function there is unknown.

**Results:**

Using a transgenic zebrafish line and immunofluorescence, we show that *Rag1 *is expressed and translated in a subset of olfactory sensory neurons (OSNs). Neurons expressing GFP under the *Rag1 *promoter project their axons to the lateral region of the olfactory bulb only, and axons with the highest levels of GFP terminate in a single glomerular structure. A subset of GFP-expressing neurons contain Gα_o_, a marker for microvillous neurons. None of the GFP-positive neurons express Gα_olf_, Gα_q _or the olfactory marker protein OMP. Depletion of RAG1, by morpholino-mediated knockdown or mutation, did not affect axon targeting. Calcium imaging indicates that amino acids evoke chemotopically organized glomerular activity patterns in a *Rag1 *mutant.

**Conclusion:**

*Rag1 *expression is restricted to a subpopulation of zebrafish olfactory neurons projecting to the lateral olfactory bulb. RAG1 catalytic activity is not essential for axon targeting, nor is it likely to be required for regulation of odorant receptor expression or the response of OSNs to amino acids.

## Background

Animals possess a number of chemosensory systems that enable them to perceive diverse stimuli in the environment. One such system is the olfactory system, which detects chemicals by a large number of olfactory sensory neurons (OSNs) in the nose. In mammals, each OSN expresses a single allele of one odorant receptor [1] on a dendrite that is exposed to the external world, and on an axon terminal that extends into the brain [2]. The projection of OSNs is highly ordered: all neurons expressing a given receptor converge to the same region in the ipsilateral olfactory bulb [3-5], terminating in a single glomerulus, i. e., a spherical area of dense synaptic neuropil. Guidance of axons is determined by a combination of factors, including the odorant receptors [6,7]. As a result of this well-ordered projection, chemical information is presented to the brain as spatial activity patterns across the array of glomeruli in the olfactory bulb [8,9].

Olfactory sensory neurons are morphologically diverse, consisting of ciliated, microvillous and crypt neurons. In fish, there appears to be some correlation between neuronal morphology, receptor class, G-protein type, and ligand spectrum [10,11]. Ciliated OSNs express receptors similar to those found in the main olfactory system of mammals express the Gα_olf _subunit and respond to amino acids or nucleotides. Microvillous neurons, on the other hand, express receptors from the V2R family found in the vomeronasal system of mammals, Gα_o_, Gα_q _or Gα_i-3_, and respond to amino acids or bile acids. In mammals, microvillous neurons detect pheromones, but can also respond to other odors [12]. Crypt neurons, which have a distinct rounded morphology, also contain Gα_q _in the apical region. Different classes of OSNs project to different regions of the olfactory bulb [10,13].

The recombination activation gene, *Rag1*, is expressed in the olfactory epithelium of mice and zebrafish [14,15]. The *Rag1 *gene, together with *Rag2*, is thought to have entered the genome of an ancestral organism 450 million years ago, soon after the divergence of jawed and jawless vertebrates; both genes have remained adjacent to one another throughout evolution. Acting together, RAG1 and RAG2 proteins function similarly to bacterial transposases such as Tn10 and are able to cleave DNA in a sequence-specific manner [16]. They mediate V(D)J recombination, and are thus responsible for the generation of antibodies and T-cell receptors [17]. As a result, each *Rag1 *expressing immune cell has a different identity, characterized by a permanent change to its genome, as well as the proteins expressed on its surface. Mutations in *Rag1 *[18] or *Rag2 *[19] lead to immunodeficiency.

The function of *Rag1 *in the olfactory system is unclear, although there has been some speculation that DNA rearrangement could be involved in odorant receptor expression [20]. As a step towards understanding the role of *Rag1*, we have initiated a study using the zebrafish, a model system with a relatively well-characterized olfactory system [21-23]. The transparency of the zebrafish larva enables gene expression analysis at single cell resolution, while genetics and morpholinos provide tools for assessing gene function using anatomical or physiological methods. Our results suggest that RAG1 protein is present in a subset of OSNs, including some microvillous olfactory neurons, but is not required for axon targeting.

## Results

### *Rag1 *expression in the zebrafish olfactory system

**Figure 1:**
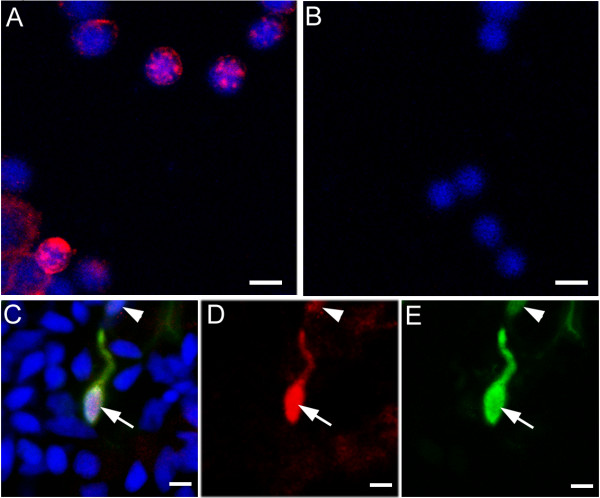
**Immunofluorescent labelling of RAG1. **(A) Isolated zebrafish thymocytes, labelled with the antibody to RAG1 (in red) and DAPI (blue). (B) After pre-absorption with the peptide used for immunization, no labelling was detected. (C-E) Double-label of olfactory epithelial cells isolated from a *Rag1*:*GFP *transgenic fish. RAG1 protein (C, D; red) is present in the GFP-positive (C, E; green) neurons (arrow and arrowhead). Neurons with high (arrow) or low (arrowhead) GFP levels contain RAG1. DAPI (C; blue) is used to stain nuclei. Bar = 5 μm.

Using a transgenic zebrafish line in which the coding sequence of *Rag1 *within a PAC was replaced with GFP, Shuo Lin and colleagues have reported the expression of *Rag1 *in olfactory sensory neurons [15]. To confirm the fidelity of GFP expression, double-labelling with antibodies to zebrafish RAG1 and GFP was carried out. The specificity of the RAG1 antibody was first tested by preabsorption and immunofluorescence on thymocytes (Fig. 1A, B). Olfactory neurons from 4 day-old *Rag1*:*GFP *fish were then isolated, fixed and doubled-labelled. In these cells, two classes of GFP expression was seen – bright and dim. An overlap of RAG1 (red) and GFP (green) immunofluorescence (Fig. 1C–E) was observed. From 3 double-labelling experiments, 100 out of 105 cells with high levels of GFP were found to be RAG1-positive, with label predominantly in the nucleus. 91 out of 102 cells that expressed a lower level of GFP were also RAG1-positive. These observations establish that RAG1 protein is present in olfactory neurons, and that GFP expression in the transgenic line can serve as an indicator of endogenous RAG1.

**Figure 2:**
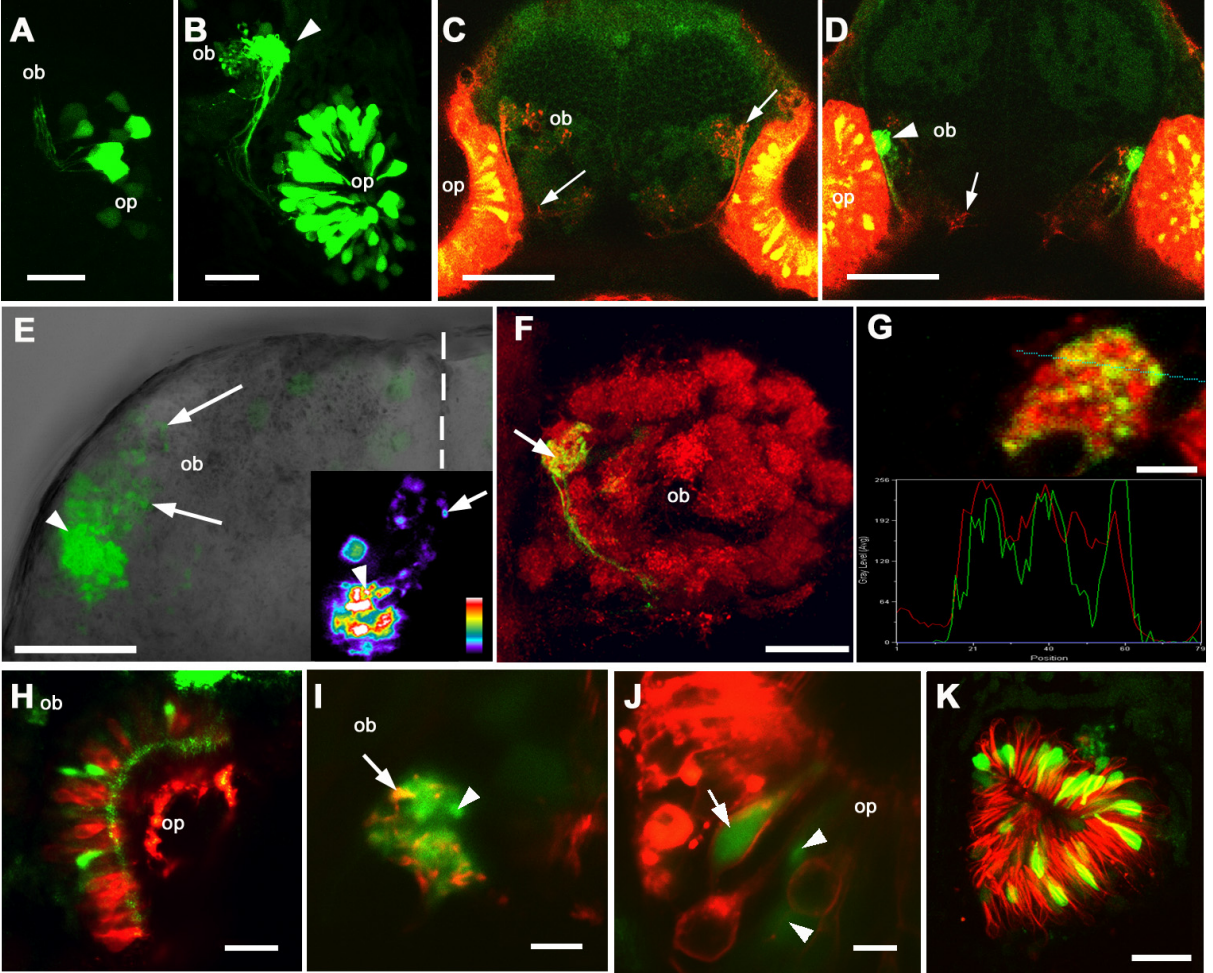
**Expression of *Rag1 *in zebrafish olfactory system. **(A) At 22 hours post-fertilization, a few olfactory sensory neurons express GFP under the *Rag1 *promoter. (B) At 3 dpf, fluorescing axons have reached the bulb. A single target (arrowhead) is innervated by brightly labelled axons. (C, D) Frontal view of an 8-day old larva, with DiI labeling of olfactory sensory neurons (red) and Bodipy labeling of inter-cellular spaces (dim green). (C) A shallow section of the labelled forebrain, with DiI-labelled olfactory axons visible (arrows). In a deeper section (D) the GFP-containing axons (arrowhead) can be seen, along with other DiI-labelled axons (arrow). (E) Dorsal view of an isolated forebrain from a 4 day-old fish, showing the left olfactory bulb. Anterior is to the top and the midline is indicated by the dotted line. Strong GFP fluorescence is seen in axon terminals in a single region of the lateral bulb (arrowhead), while axons with lower levels of GFP innervate other regions of the lateral bulb (arrows). The inset shows one optical section, colour-coded according to fluorescence intensity. Termini with high (arrowhead) and low (arrow) intensity are indicated. (F) Frontal view of glomerular structures in the olfactory bulb of a 4-day fish, labelled with an antibody to synaptic vesicles. Only one lateral structure is innervated by OSNs with strong GFP expression (arrow). Lateral is to the left, while dorsal is to the top. (G) A single optical section through the glomerular target containing GFP-expressing neurons. The marker for synaptic vesicles (red) and GFP appear to co-localize, as indicate by the linescan. (H) An olfactory pit labeled with DiI. The GFP-expressing cells (green) have not taken up DiI (red). (I, J) A Di8ANEPPQ-labeled olfactory system of a *Rag1*:*GFP *transgenic fish. In the olfactory bulb (I), some axons with strong GFP expression (green) are also labeled with Di8ANEPPQ (red; arrow), whereas others are not (arrowhead). (J) In this section through the olfactory epithelium, a GFP-expressing neuron was labeled with Di8ANEPPQ (arrow), whereas two others were not (arrowheads). (K) The olfactory pit of a fish transgenic for *Rag1:GFP *and *omp:tauDsRed*. GFP expressing cells (green) are distinct from those labeled with DsRed. Panels A, B, E, F and K are projections reconstructed from Z stacks. ob: olfactory bulb; op: olfactory pit. Bar = 20 μm (A, B, E, F, H, I, K); 50 μm (C, D,); 5 μm (G, J).

We imaged the *Rag1:GFP *fish at high resolution to determine in more detail the expression of *Rag1 *in the zebrafish olfactory system. GFP was first detectable at 22 hours post-fertilization, and both bright and dim cells could be seen in the olfactory epithelium (Fig. 2A). Initially, only short axons were visible. By 72 hours, the number of fluorescing cells increased and axons reached the olfactory bulb (OB; Fig. 2B). Fluorescing neurons projected axons to the lateral region of the OB (Fig. 2C–E). Intense labelling was detected in one target (Fig. 2E), which is a glomerular neuropil structure, as identified by labelling with the synaptic vesicle marker SV2 [24] (Fig. 2F). Synaptic vesicles co-localize with the termini of GFP-positive axons in this glomerular structure. (Fig. 2G).

When the fluorescent lipophilic dye DiI was placed in the fish water, many OSNs took up the dye (Fig. 2C, D). However, few (13.8%; n = 58) strongly GFP-expressing neurons were labelled (Fig. 2H). This suggests a low accessibility to the external environment within this subpopulation. With Di8ANEPPQ, a lipophilic dye with higher solubility, a higher proportion (53.8%; n = 26) of strong GFP-positive cells were labelled (Fig. 2I, J), confirming that they do have some access to the water. We then examined *Rag1 *expression in fish expressing tauDsRed under the control of a promoter fragment of the olfactory marker protein (OMP) gene [25]. In this line, tauDsRed is expressed in most OSNs except for a subpopulation projecting to a lateral area in the developing OB [25]. In *Rag1*:GFP, *omp:tauDsRed *double transgenic fish, GFP was detected in OSNs that were tauDsRed-negative (Fig. 2K). The GFP-positive neurons appeared to have a shorter cell body than those expressing tauDsRed.

**Figure 3:**
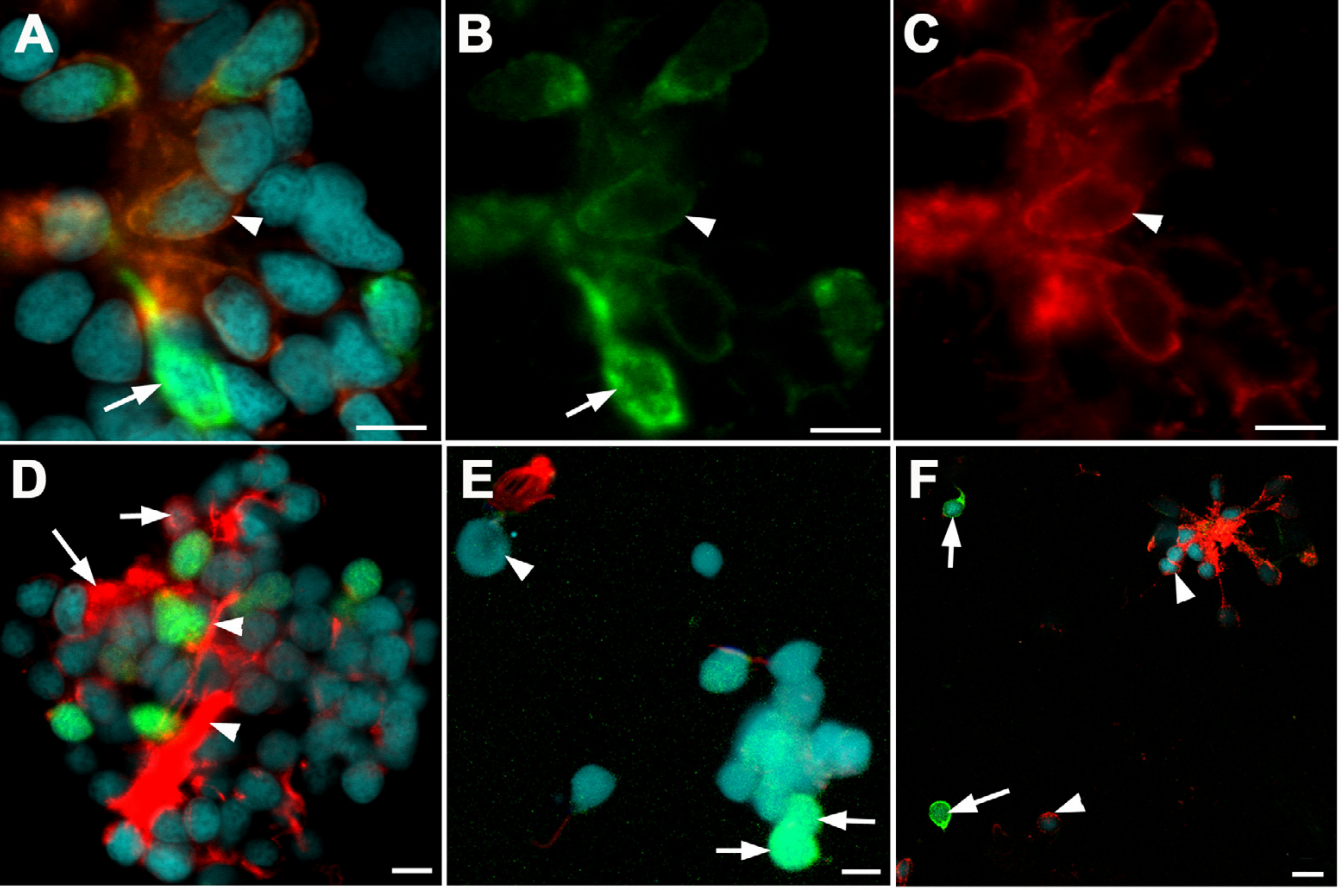
**Expression of G alpha subunits in larval olfactory neurons.**(A-C) Gα_o _(red fluorescence, C) is present in a subset of *Rag1*-expressing cells (green fluorescence, B; arrowheads) isolated from the olfactory epithelium of a *Rag1:GFP *zebrafish. A cell with bright GFP (arrow), however, does not contain Gα_o_. (D) Gα_q_-positive neurons (arrows) do not express GFP, and vice-versa. Axons are strongly labelled (arrowhead). (E) A crypt cell (arrowhead), with Gα_q _label in the dendrites, lacks GFP. Two cells with bright GFP (arrows), in contrast, lack Gα_q _label. (F) Gα_olf _label, seen here in axons and some cell bodies (arrowheads), was not detected in cells strongly expressing GFP (arrows). Bar = 5 μm (A-E); 10 μm (F).

When labelled with antibodies to different G-alpha subunits, cells with strong GFP expression were Gα_olf_, Gα_o _and Gα_q_-negative (Fig. 3). Crypt cells or other neurons that were labelled by the Gα_q _antibody were GFP-negative (Fig. 3D, E). 36 out of 85 neurons containing Gα_o _expressed GFP at low levels (Fig. 3A–C). The remaining cells had no GFP. An additional 13 cells had low GFP expression, but did not express Gα_o_. These observations indicate that the GFP-expressing cells are heterogenous in nature.

**Figure 4:**
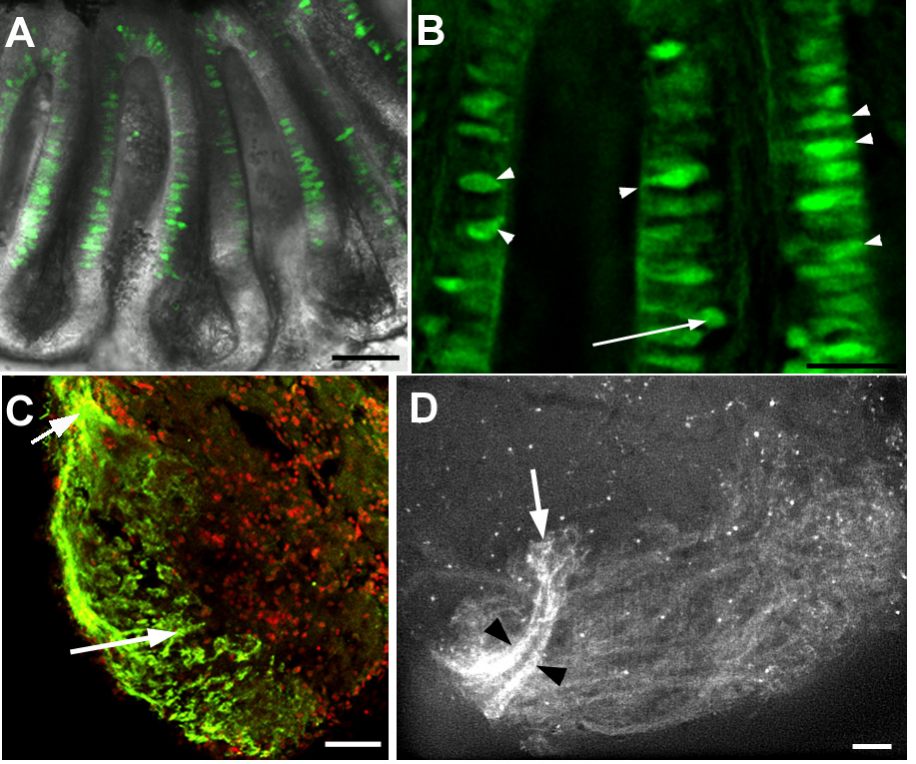
**Expression of *Rag1 *in the adult zebrafish olfactory system.**(A) An olfactory rosette isolated from a 1.5 year-old adult. GFP-expressing cells are located in the sensory region of the lamella and midline raphe. Most cell bodies are located apically. (B) High magnification of lamella from another olfactory rosette, also from a 1.5 year-old adult. GFP-expressing cells have differing morphologies. Many have the cell body close to the apical surface (arrowheads). The arrow indicates one neuron with a deep cell body. (C) Anti-GFP immunofluorescence on a horizontal section through the olfactory bulb of an adult. Label is detectable only in olfactory sensory neurons innervating the lateral bulb. The incoming olfactory nerve is visible at the upper left (arrow). Anterior is to the top-left; lateral to the bottom-left. Nuclei are labelled with propidium iodide (red). (D) Lateral view of an olfactory bulb dissected from a 1.5-year-old *Rag1:GFP *male. Axons innervate a large portion of the lateral bulb, but axons with the highest levels of GFP (black arrowheads) form two bundles that innervate a single region (arrow). Other glomeruli are innervated by dimmer axons. Bar = 50 μm (A, C, D); 20 μm (B).

In the adult, GFP expression could still be detected in the olfactory system. Most GFP-positive neurons had their cells bodies close to the apical surface of the olfactory epithelium, with dendrites of intermediate length; a few cells, however, had their soma deep in the epithelium (Fig 4A, B). GFP-positive axons project to the lateral olfactory bulb only, as can be seen in dissected forebrains, or using immunofluorescence on sections (Fig. 4C). A subset of axons projecting to one target in the lateral bulb contained particularly high levels of GFP (Fig. 4D).

### The effect of RAG1 knockdown on axon targeting

**Figure 5:**
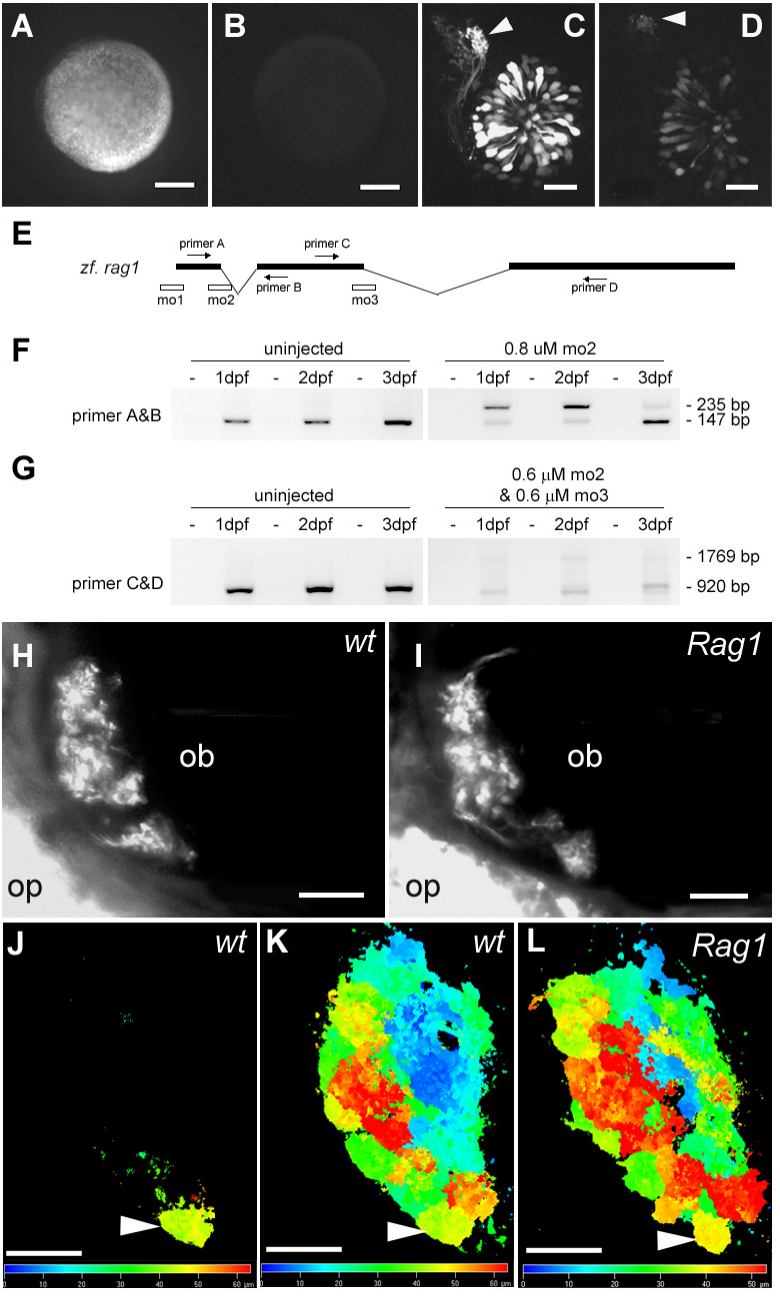
**The effect of RAG1 depletion on the olfactory projection. **(A) An embryo injected with mRNA encoding the 5' end of *Rag1 *fused to EGFP. (B) An embryo co-injected with the *Rag1*-EGFP fusion mRNA and mo1. (C) Olfactory neurons labelled with GFP under the *Rag1 *promoter, with brightly labeled axons projecting to a single target (arrowhead), at 3 dpf. (D) In a transgenic embryo injected with mo1, axons still project to the same target (arrowhead), but the intensity of GFP fluorescence is reduced. (E) A schematic diagram of the *Rag1 *gene, showing the location of morpholinos and primers that were used to analyse morpholino-injected fish. (F) RT-PCR on control or mo2 injected embryos. Abnormal splicing occurs in the morpholino-injected fish, leading to a premature stop codon, as indicated by sequencing of the upper band. (G) RT-PCR after injection of a mixture of *Rag1 *mo2 and mo3, showing loss of the normal transcript. (H, I) A subset of Di8ANEPPQ-labeled olfactory sensory neurons in 7-day old wild type and *Rag1 *mutant fish. Axons innervate all target structures detectable in this optical plane in the mutant. (J-L) SV2-labelled 4 day-old *Rag1:GFP *transgenic (J, K) and *Rag1 *mutant (L) forebrains, shown in dorsal view. The images are colour-coded according to depth. The glomerulus innervated by the strong GFP-positive neurons (J) is indicated by the arrowhead. Bar = 100 μm (A, B); 20 μm (C, D, H – K). The colour bars in J and K indicate depth. Embryos in panels H-L are shown in dorsal view, with anterior to the left.

The expression of GFP in a subpopulation of neurons that project to one region of the olfactory bulb raises the possibility that Rag1 could be involved in specifying the identity, and hence the axonal targeting, of these neurons. To test this idea, three morpholinos were used to knock down Rag1 in embryos. The first morpholino (mo1) targeted the start codon of RAG1 and was tested using a fusion of the 5' end of RAG1 to GFP. Injection of mRNA for the fusion protein alone led to a strong fluorescence (Fig. 5A), whereas co-injection with the morpholino suppressed fluorescence (Fig. 5B), indicating that mo1 knocked down *Rag1 *translation. The second morpholino (mo2) was targeted to the splice donor site of the first exon (Fig. 5E). Injection of this morpholino led to aberrant splicing (Fig. 5F), resulting in an mRNA with a premature stop codon (data not shown). The third morpholino (mo3) was designed to the donor site of the second exon (Fig. 5E), and this resulted in loss of the normal transcript (not shown). Splice donor morpholinos were tested because the use of internal ATGs, which has been reported for some forms of Omenn's syndrome [26], might obscure the effects of mo1.

Injection of mo1 caused a reduction in the level of GFP driven by the *Rag1 *promoter. This occurred despite there being an 8-nucleotide mismatch in the transgene (since GFP replaces the *Rag1 *coding sequence), presumably because the mismatch is contiguous and exclusively at one end of the morpholino. The convergence of brighter axons to one lateral target persisted (Fig. 4C, D; n = 15). Aside from the decrease in GFP expression, no other defect could be detected. With mo2, injections of 0.8 μM caused slight non-specific morphological abnormalities, such as small eyes. Injection with 0.8 μM mo3 caused stronger abnormalities. Less abnormality was seen when a mixture of 0.6 μM mo2 and 0.6 μM mo3 was injected. This led to a dramatic loss of normally spliced *Rag1 *mRNA (Fig. 5G). Injection of either mo2 or mo3 or the mixture did not cause any defect in the targeting of the GFP-expressing neurons (n = 50 embryos each). Hence strong *Rag1 *expression is not required for innervation of the lateral target.

As an additional step in analysing the role of *Rag1 *in establishing the olfactory projection, a line carrying a point mutation, leading to a STOP codon within the RAG1 catalytic domain [27], was examined. The entire projection was labeled with the lipophilic tracer Di8ANEPPQ. No obvious difference could be seen between mutants and wild types (Fig. 4H, I). To further analyse mutants, the forebrains of 4 day-old larvae were labelled with the SV2 antibody. The lateral neuropil structure that is innervated by neurons with strong GFP expression could be visualized in mutants, as in wild types (Fig. 5 J-L). Together with the morpholino data, these observations indicate that Rag1 is not required for pathfinding of OSN axons to the olfactory bulb, or for establishment of glomeruli.

### Odor-evoked activity maps in the *Rag1 *mutant

The spatial organization of OSN axons in the olfactory bulb and their functionality was further examined by calcium imaging of odor-evoked activity maps in 3 month-old fish. OSN axons were loaded with the calcium indicator, Calcium Green-1-dextran. Odor-evoked activity in OSN axons leads to calcium influx at axon terminals, causing a localized change in indicator fluorescence. This method allows for the optical detection of odor-evoked activity selectively in OSN axons within glomeruli [9]. In wild type fish, amino acids evoke activity predominantly in the ventro-lateral region of the OB that contains densely packed, small glomeruli [9]. Within this region, clusters of glomeruli can be identified by their response to amino acids sharing particular chemical features (e. g., a short neutral side chain, a long neutral side chain, or a basic side chain). Response patterns vary considerably between individuals, but functionally defined clusters are found at similar relative positions along the anterior-to-posterior axis in different individuals, thus establishing a conserved chemotopic map [9].

**Figure 6:**
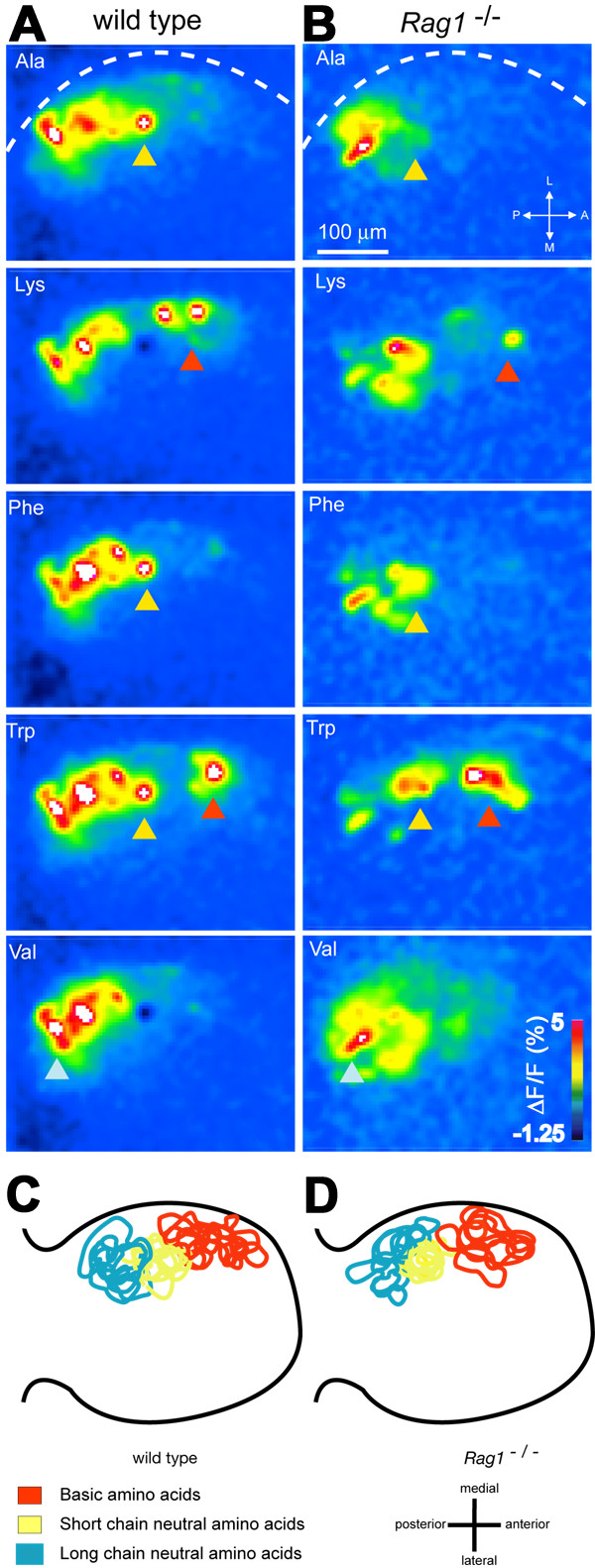
**Chemotopic organization of glomerular activity patterns in wild type and Rag1 mutant fish. **(A) Glomerular activity patterns evoked by different amino acid stimuli (10^-5 ^M) in the ventro-lateral OB of a wild type fish. Changes in fluorescence intensity report activity of OSN axon terminals and are colour coded. Dashed line depicts lateral edge of the OB. Arrowheads indicate conserved clusters of glomeruli with defined response properties. (B) Glomerular activity patterns evoked by the same stimuli in a *Rag1 *mutant fish. The same general areas of the bulb show a response. The difference in the intensity seen here between wild type and mutants is not statistically significant. (C) Overlay of positions of identifiable glomerular regions in wild type of the line from which *Rag1 *mutants were derived (n = 7). Regions were outlined manually in activity maps in each fish. Outlines from different individuals were centered on the central cluster (yellow). (D) Overlay of cluster positions determined in 10 *Rag1 *mutant fish.

The distribution and magnitude of response patterns evoked by a diagnostic set of L-amino acids with different chemical features (Met, Ala, Val, Lys, Phe, His, Trp; each 10^-5 ^M) were generally similar in wild type and *Rag1 *mutant fish (Fig. 6A, B). However, in both wild type and *Rag1 *mutants, the fine structure of these activity patterns varied between individuals. To examine the chemotopic organization in more detail, we therefore defined three glomerular regions by characteristic response properties, as done previously to identify chemotopically organized glomerular clusters [9]. The first region was defined by responses to the basic amino acid, Lys, and partially to Trp (Fig. 6A, B; red arrowheads), the second region was defined by responses to all short-chain neutral stimuli (Phe, Trp and Ala; Fig. 6A, B; yellow arrowheads), and the third region was defined by the response to the long-chain neutral amino acids, Val and Met (Fig. 6A, B; light blue arrowhead). Regions with these functional properties were identifiable in each wild type and *Rag1 *mutant fish. Their spatial arrangement was analyzed by overlaying the outlines of the regions from different individuals. In both wt (n = 7) and *Rag1 *mutant (n = 10) fish, the three clusters occurred in a characteristic sequence along the anterior-to-posterior axis (Fig. 6C, D), as described previously [9]. Hence, the chemotopic organization of glomerular response maps was comparable in wild type and *Rag1 *mutant fish. Other response properties, such as wide-spread responses to food extracts [28], were also similar between wild types and *Rag1 *mutants (not shown). We also quantitatively analyzed the similarity relationships between activity patterns evoked by different stimuli using correlation and factor analysis [9] and observed no obvious differences between wild type and *Rag1 *mutant fish. Hence, physiological responses of OSNs to amino acids and their chemotopically organized projections to the OB were not detectably altered in *Rag1 *mutants.

## Discussion

To delineate possible functions of *Rag1 *in the zebrafish olfactory system, we have examined its expression pattern in detail and examined the effect of RAG1 loss. Immunofluorescence using an antibody to zebrafish RAG1 indicates that the protein is present in OSNs. Imaging of a transgenic line, which is a sensitive method to monitor gene expression in neurons [29], suggests that *Rag1 *is expressed only in a subset of OSNs. Some of the neurons expressing *Rag1 *are likely to be microvillous neurons, as indicated by their morphology and Gα_o _expression. Neurons expressing GFP project to the lateral bulb, which is a region of the bulb known to contain termini of amino-acid sensitive OSNs [9]. Their projection pattern appears to be complementary to neurons expressing a reporter under the OMP promoter, as these project to the medial bulb [30]. *Rag1 *expression thus appears to mark a subset of distinct OSNs. These neurons are likely to be heterogenous, given that not all of them express Gα_o_.

A feature of the *Rag1*:GFP transgenic line analyzed here is that one glomerular target in each bulb is more brightly labelled than other targets. One interpretation of this observation, given that some cell bodies, axons and termini are brighter than others, is that neurons expressing GFP at high levels all have a particular identity and project to a single target. These neurons do not express any of the markers tested, possibly because of their distinct identity. Alternatively, they may not be fully mature when GFP is expressed strongly. This would be consistent with the finding that not all bright neurons take up DiI or Di8ANEPPQ from the external environment, which would be the case if their dendrites were immature. These neurons may eventually have reduced levels of GFP, and express Gα_o_. It should be noted that high levels of GFP expression cannot be a feature of all immature OSNs. If this were the case, it would be expected that there would be bright neurons projecting to all glomeruli; this was never seen. Although we cannot yet make any firm conclusions as to why some axons are brighter than others, their reproducible targeting can serve as a useful marker in genetic screens or in physiological analysis.

In the olfactory system, sensory neurons are distinguished from one another on the basis of odorant receptor expression, and targeting to a particular glomerulus is dependent on the odorant receptor [6], amongst other axon guidance cues. It has been suggested that odorant receptor expression could be regulated by a mechanism similar to V(D)J recombination, given the genomic organization of the receptors. We found that axon targeting and chemotopic activity maps in the OB were not affected by knockdown of *Rag1 *or by a mutation that abolishes the catalytic activity of *Rag1*. Hence, it appears that RAG1 does not have a role in defining the identity of OSNs. Recent cloning experiments show that the genome of mammalian OSNs is not rearranged [31,32]. Those experiments were carried out with neurons expressing receptors of the OR class, i.e. those expressing Gα_olf_, which is probably not expressed in *Rag1*-positive OSNs in zebrafish. The observations described here suggest that this conclusion also holds for other olfactory neurons.

RAG1 contains the active site for V(D)J recombination [33,34] and can cleave DNA in vitro on its own, although it does so with low efficiency [35]. Once DNA has been cleaved, RAG1 can bind to the broken ends and serve a protective role [36]. Given the relatively high rate of DNA breaks in neurons [37], one possibility is that RAG1 protects the genome in some neurons. However, this view does not explain why only a subset of neurons expresses *Rag1*. Another function of RAG1, provided by the RING-finger, is as an E3 ligase [38], raising the possibility that this activity may be required in a subset of receptor neurons. Additionally, RAG1 can independently act as an endonuclease [39]. It is apparent that RAG1 can carry out several functions, but it is unknown at present whether the expression of *Rag1 *in the olfactory epithelium reflects any of these abilities. The expression described here suggests that any function of *Rag1 *must be restricted to a subset of OSNs only.

## Conclusion

This study demonstrates that *Rag1 *is expressed and translated in a subset of zebrafish olfactory neurons projecting to the lateral olfactory bulb. Some of these neurons express a marker of microvillous neurons. *Rag1 *does not appear to be essential for axon targeting or receptor expression.

## Methods

### RAG1 antibody

A rabbit polyclonal antibody against the C-terminus of zebrafish Rag1 (amino acids 1042~1057) was generated by ZYMED Laboratory Inc. The peptide antigen (CEETPEEADNSLDVPDF) was synthesized by Tufts University Core Facility. To test for specificity, the antibody was incubated overnight at 4°C with the peptide at a concentration of 66.6 μg/ml. After a 30 minute spin at 16 000 g, the supernatant was used for labelling thymocytes, which had been dissected from freshly killed 2 week-old zebrafish using tungsten needles.

### Immunofluorescent labelling of olfactory neurons

4 day-old *Rag1:GFP *fish were fixed in 4% PFA-PBS for 10 minutes. The olfactory epithelium was dissected out in Ringer's solution and placed on Superfrost/Plus slides using a mouth pipette (Sigma A5177). The olfactory epithelium was allowed to semi-dry and adhere to the slide. A drop of Ringer's solution was added and a cover slip was used to squash the epithelium gently, thus dispersing the cells and allowing them to adhere to the slide. The cover slip was gently removed and the cells were re-fixed in 4% PFA-PBS for another 5 minutes. They were then rinsed with PBS and permeabilised with 1% Triton X-100. The following antibodies were used: anti-zf RAG1 (rabbit, 1:200), anti-GFP (mouse, 1:50; Molecular Probes), anti Gα_o _(guinea pig, 1:200), Gα_q _and Gα_olf _(both rabbit, 1:200; Santa Cruz Biotech). The Gα_o _antibody [40] was made to a region of the protein that is 100% conserved between *Drosophila *(residues 345–354) and zebrafish. For detection, 1:500 Cy3 anti-guinea pig, 1:300 AlexaFluor 568 anti rabbit and 1:300 AlexaFluor 488 anti mouse (Molecular Probes) were used. Nuclei were stained with 100 ng/ml DAPI. Cells were imaged with confocal microscopy. At least 50 neurons with strong GFP expression were analysed for each G-alpha subunit labelling.

### Immunofluorescent labeling of glomeruli

The brain of 4-day old Rag1:GFP fish larvae was dissected out in Ringers and fixed for 1 hour in 4% PFA-PBS. Wholemount antibody labelling was carried out using standard procedures, using the SV2 antibody (Developmental Studies Hybridoma Bank) at 1:500 dilution, and the AlexaFluor 546 goat anti-mouse antibody (Molecular Probes) at 1:500 dilution.

### Immunostaining of cryo-sectioned tissue

The brains of 3 month-old *Rag1:GFP *transgenic fish were dissected out in PBS and fixed in 4% PFA, then embedded in tissue freezing medium (Jung) and sectioned. The sections were incubated in rabbit anti-GFP (1:200) followed by AlexaFluor 488 anti-rabbit (1:300). Nuclei were stained with propidium iodide.

### Constructs

**5'RAG1-EGFP: **A 350 bp 5' *Rag1 *DNA fragment was amplified by RT-PCR with primer Rag1a: CTCTCAATTCATAAAAAATAAATCTTAC and Rag1b: GGTCCACTCTCCCTCGAG, digested with Hae III and inserted into Smal I site of pEGFP-N1 (Clontech). For in vitro transcription of the fusion mRNA, the 5'RAG1-EGFP fragment was cut out and cloned into pCS2 [41].

### Imaging

Live zebrafish embryos and larvae were embedded in 1.5 % low-melting temperature agarose (BioRad) and imaged with Zeiss LSM 510 laser scanning confocal microscopy, using 40 × (0.8 NA) or 63 × (1.2 NA) water immersion objectives. Isolated olfactory bulbs from larvae were imaged with a 20 × (0.5 NA) water immersion objective.

Dissected olfactory bulbs and rosettes from adults were imaged using widefield fluorescence, with 10x or 40xW objectives. Images were deconvolved using AutoDeblur (AutoQuant Inc.)

### Lipophilic tracing of olfactory neurons

A saturated stock solution of DiI or Di8ANEPPQ (Molecular Probes) was prepared in ethanol. DiI was diluted 1:1000 in E3, while Di8ANEPPQ was diluted 1:5000, just before use. Larvae were placed in this solution in mesh baskets for 3 minutes, and then rinsed several times in fresh E3. Bodipy labelling was carried out as described [21].

### Morpholino injection

Morpholinos (Gene-Tools) were diluted in H_2_O and injected into one-cell stage embryos. 50~100 embryos were injected with each oligo, with single morpholino concentrations ranging from 0.2 to 1.0 μM. Injection volume was approximately 1 nl. The following morpholinos were used:

**mo1**: 5'-TTCTCCATGGCGTCAGCTTATTCTC-3' (targets the start codon). **mo2**: 5'-TATTATACTCACTTGAGAAGATTCA-3' (targets the donor site of the first intron). **mo3**: 5'-TCTTGGCAGTACCTTGCATCATTGC-3'(targets the donor site of the second intron).

### Genotyping of the *Rag1 *mutant

Allele specific PCR [42] was carried out using the following primers:

Rag1f1: CACTggCCCATgCTCCgATAgACC;

Rag1r1: TCCGGGGCACAGGCTATGATGAGAA;

Rag1wtr: GCTTAGCAGAAACACCTTTGACTCg;

Rag1mutr: GCTTAGCAGAAACACCTTTGACTCa.

### Imaging of activity in OSN axon terminals

OSNs of adult zebrafish were loaded with Calcium Green-1 dextran (10 kD; Molecular Probes) and imaged as described previously [9]. Briefly, 6–8% of the dye in 3mM NaCl and 0.1%Triton X-100 was applied to each naris while the fish was anesthetised with 0.01% MS-222. Following a 5-minute incubation, the dye was washed away and the fish were allowed to recover. 3–5 days post labelling, the brain and nose were dissected, mounted upside-down in a custom-made perfusion chamber, and superfused with teleost artificial cerebrospinal fluid (ACSF). Amino acid solutions and extracts of commercially available fish foods were applied through a constant stream of ACSF to one naris using a computer controlled, pneumatically actuated HPLC injection valve (Rheodyne) as described [9,28].

### Ca^2+ ^imaging

Fluorescence in the olfactory bulb was recorded using a CCD camera (CoolSnapHQ; Photometrics) mounted on a custom-built upright epifluorescence microscope equipped with a 20x lens (NA 0.95; Olympus) [43]. Fluorescence was excited with a stabilized 150 W Xe arc lamp attenuated to 1.5% of full intensity by neutral density filters to minimize bleaching and phototoxicity. Images were binned to 87 × 65 pixels, acquired at 2 – 10 Hz, and digitized with 12 bits. Each pixel value was converted to a value representing the relative change in fluorescence (ΔF/F) after stimulus application. The baseline fluorescence (F) was calculated by averaging the frames before stimulus onset. Response maps were obtained by averaging ΔF/F frames over 2–4 s after response onset and mild spatial filtering.

## Authors' contributions

FB documented the specific targeting of *Rag1*-expressing neurons in embryos and carried out morpholino experiments. SB conducted the immunofluorescence. SB and EY performed the calcium imaging, with critical help from RWF. RWF designed and analysed the physiological experiments, and helped draft the manuscript. SJ conceived the study, carried out the glomeruli labelling and imaging of adults, and drafted the manuscript.
